# The vitamin D-cardiovascular nexus unraveling mechanistic promise and trial reality

**DOI:** 10.1186/s12986-026-01108-1

**Published:** 2026-03-17

**Authors:** Hui Xu

**Affiliations:** Gaozhao Street Community Health Service Center, Xiuzhou District, Jiaxing, 314015 China

**Keywords:** vitamin D, cardiovascular diseases, molecular mechanisms, randomized controlled trials, precision nutrition

## Abstract

**Background:**

Epidemiological data consistently associate low vitamin D status with elevated cardiovascular risk, yet causal mechanisms remain contested.

**Methods:**

This review synthesized evidence through a protocol-guided approach, with database searches (PubMed/Embase/Web of Science; 2010–2024) focused on RCTs, mechanistic studies of cardiovascular pathways, and high-quality meta-analyses (AMSTAR-2 score ≥ 8).

**Results:**

Mechanistic investigations consistently demonstrate vitamin D’s pleiotropic cardiovascular actions, including suppression of the renin-angiotensin-aldosterone system, enhancement of endothelial nitric oxide bioavailability, and attenuation of vascular inflammation and fibrosis—effects validated in human models. In contrast, large-scale randomized trials and their meta-analyses have uniformly failed to demonstrate significant cardiovascular benefit from vitamin D supplementation in the general population. This discordance appears largely attributable to methodological limitations in trial design, particularly the inclusion of predominantly vitamin D-sufficient participants and the absence of stratification for baseline deficiency status or genetic determinants of vitamin D metabolism. Notably, post-hoc analyses suggest potential signal enrichment in cohorts with severe baseline deficiency, though these observations require prospective validation.

**Conclusions:**

Current evidence does not support population-wide vitamin D supplementation for cardiovascular risk reduction. The persistent mechanistic-clinical disconnect underscores fundamental gaps in trial methodology rather than invalidating biological plausibility. Future research must prioritize precision nutrition frameworks that integrate baseline vitamin D status, dynamic response biomarkers, and genomic profiling to identify subgroups where supplementation may confer meaningful benefit. Until such evidence emerges from rigorously designed trials, clinical efforts should focus on correcting documented deficiencies in high-risk metabolic phenotypes, with cardiovascular outcomes regarded as potential secondary benefits rather than primary therapeutic targets.

## Introduction

 Cardiovascular disease (CVD) remain the leading cause of global morbidity and mortality, and early intervention targeting modifiable risk factors (e.g., blood pressure) represents a key strategy for alleviating the global burden of CVD [1]. Historically recognized for its role in skeletal homeostasis, vitamin D has garnered significant investigative interest in cardiovascular medicine over recent decades. Recent evidence underscores that beyond its classical role in skeletal homeostasis, vitamin D orchestrates a repertoire of pleiotropic effects, engaging endocrine, vascular, and immunomodulatory pathways that may collectively contribute to cardiovascular protection [2, 3].

Epidemiological studies have consistently documented an inverse, dose-dependent gradient between circulating 25-hydroxyvitamin D (25(OH)D) and the prevalence of diverse CVD phenotypes. concentrations and the prevalence of diverse CVD manifestations, including hypertension, coronary artery disease, heart failure, and stroke [4]. This epidemiological foundation, coupled with the discovery of vitamin D receptors (VDRs) and metabolizing enzymes in cardiovascular tissues, provided a compelling biological rationale for a causal relationship [5]. Consequently, correcting vitamin D insufficiency could offer a simple, cost-effective strategy for mitigating CVD risk.

A series of large, rigorously conducted randomized controlled trials (RCTs) has failed to demonstrate the anticipated cardiovascular benefits of vitamin D supplementation in the general population [6]. This striking discordance between compelling mechanistic rationale and consistently neutral clinical outcomes represents a central paradox in contemporary cardiovascular prevention. Rather than merely reiterating this gap, the present review aims to systematically dissect its multifactorial underpinnings. Potential explanations are critically examined, encompassing methodological constraints in trial design as well as the intrinsic biological complexity of vitamin D signaling across diverse physiological contexts. On this basis, a translational framework is proposed that shifts the paradigm from indiscriminate supplementation toward biomarker-guided, genetically informed, and context-dependent precision nutrition strategies.

## Mechanistic rationale: the molecular basis for cardioprotection

**Fig. 1:**
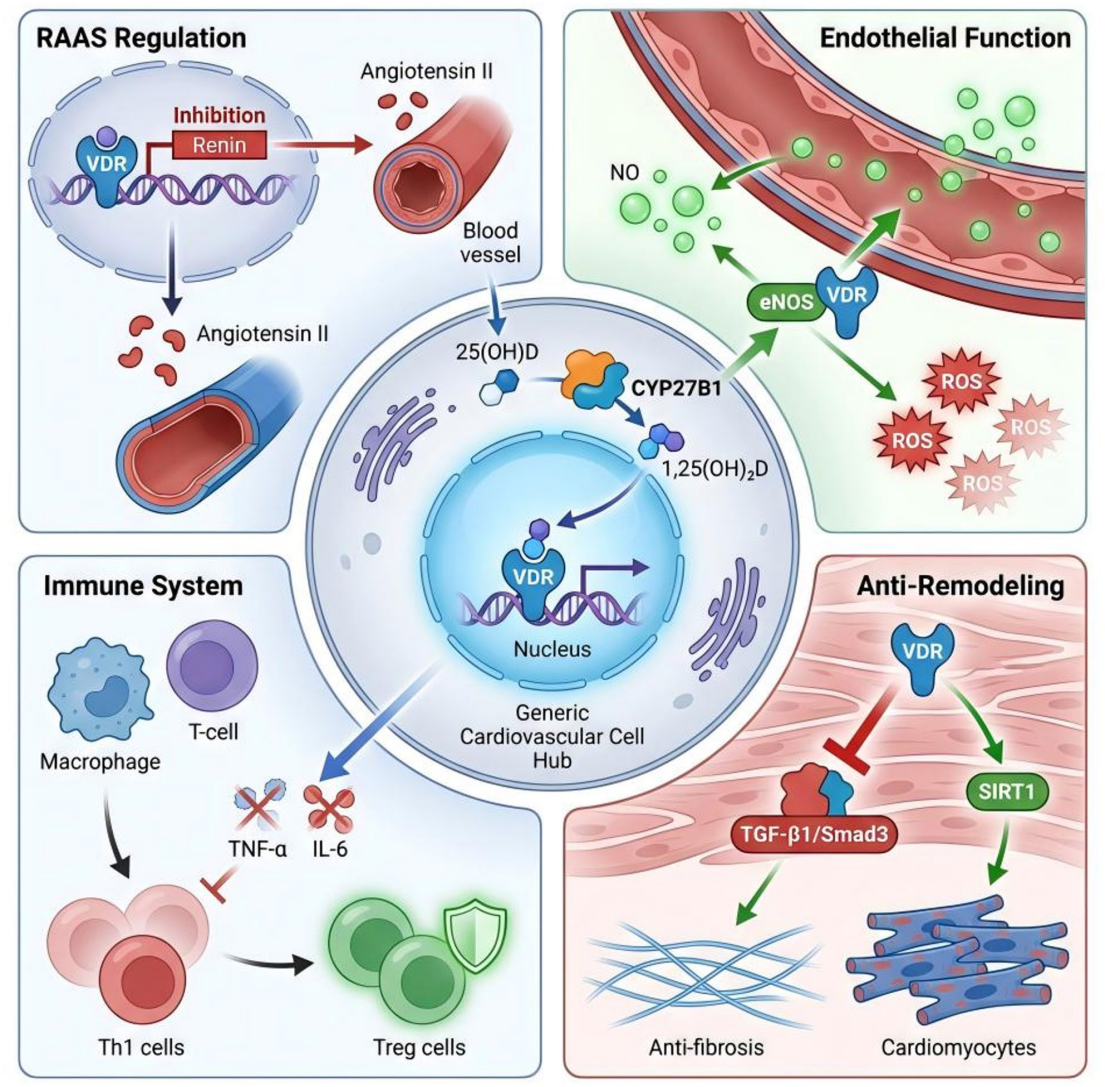
Molecular Basis for Vitamin D-Mediated Cardioprotection

The molecular basis for vitamin D’s cardioprotective potential, elucidated over the past two decades, involves a dual signaling paradigm (Fig. 1). Its pleiotropic actions on diverse cell types—including cardiomyocytes, vascular endothelial cells, and immune cells—are mediated through both genomic (via VDR-mediated transcriptional regulation) and rapid non-genomic pathways [7]. A thorough understanding of vitamin D metabolism is critical for defining its physiological functions [8]. Vitamin D activation involves two steps: hepatic hydroxylation to form 25(OH)D (the primary circulating form and clinical standard for assessing status), followed by renal hydroxylation to generate biologically active 1,25-dihydroxyvitamin D (1,25(OH)D), a secosteroid hormone. The presence of vitamin VDRs and 1-α-hydroxylase (CYP27B1) in cardiovascular tissues enables local vitamin D signaling and activation [9]. This localized system allows vitamin D to exert direct autocrine and paracrine effects on cardiac and vascular cells, independent of systemic circulation.

Emerging evidence further implicates vitamin D in the regulation of vascular calcification, a critical determinant of cardiovascular morbidity. Mechanistic studies have demonstrated that vitamin D signaling through the vitamin D receptor restrains vascular calcification by inhibiting the osteogenic transdifferentiation of vascular smooth muscle cells, an effect mediated through suppression of Wnt/β-catenin activity and downregulation of the osteogenic transcription factor Runx2, thereby extending the vasoprotective actions of vitamin D beyond classical calcium–phosphate homeostasis [10, 11].

### Regulation of the renin-angiotensin-aldosterone system (RAAS)

A principal mechanism underpinning vitamin D’s regulation of cardiovascular function is its transcriptional repression of the renin gene via VDR activation. Govender et al. demonstrated that 1,25-dihydroxyvitamin D_**3**_ exerts transcriptional repression of the human renin gene through epigenetic remodeling, characterized by reduced DNA methylation within a silencer regulatory element and consequent reinforcement of VDR-dependent suppression of renin gene expression in human cell models [12]. By serving as a fundamental check on renin synthesis, vitamin D restrains the entire RAAS, thereby limiting the production of angiotensin II [13]. This suppression mitigates key angiotensin II-mediated effects, including systemic vasoconstriction, renal sodium retention, and the stimulation of profibrotic pathways, which collectively drive hypertension and maladaptive cardiac remodeling [14]. Consequently, vitamin D deficiency disrupts this inhibitory regulation, leading to RAAS overactivation and its associated detrimental sequelae [15]. Animal models deficient in VDR or CYP27B1 consistently show elevated renin activity and hypertension, both of which are reversible with vitamin D administration [13]. These findings strongly support the role of vitamin D in regulating this pathway and suggest a mechanism for modulating human blood pressure.

### Enhancement of endothelial function and vasodilation

A cornerstone of vitamin D’s vasoprotective role is its maintenance of endothelial homeostasis. It critically upregulates endothelial nitric oxide synthase (eNOS) expression and activity, thereby enhancing nitric oxide (NO) production [16]. Concurrently, it attenuates reactive oxygen species (ROS) production, thereby reducing oxidative stress and preserving NO bioavailability [17]. This concerted action promotes vasodilation and counters endothelial dysfunction, a pivotal early event in atherogenesis. Human intervention studies confirm that vitamin D supplementation enhances flow-mediated dilation in individuals with baseline deficiency, directly supporting its role in preserving endothelial function [18]. Beyond classical nitric oxide–mediated pathways, integrity of the endothelial glycocalyx—a proteoglycan-rich surface layer that modulates mechanotransduction and limits leukocyte adhesion—has emerged as a critical determinant of vascular health, and nutritional factors including vitamin D status have been proposed to influence glycocalyx homeostasis in observational and mechanistic contexts [19].

### Anti-inflammatory and immunomodulatory effects

Chronic inflammation is a cardinal feature of atherosclerotic pathogenesis. Vitamin D counteracts this process through a bifaceted immunomodulatory role. It suppresses the production of key inflammatory cytokines, including TNF-α and IL-6, by innate immune cells such as macrophages [20]. Concurrently, it orchestrates a shift in the adaptive immune response, favoring the expansion of anti-inflammatory T regulatory (Treg) cells over pro-inflammatory T helper 1 (Th1) cells [21]. These actions may stabilize atherosclerotic plaques, reduce vascular inflammation, and potentially slow the progression of atherosclerosis [22]. Vitamin D’s anti-inflammatory properties may be particularly relevant in heart failure with preserved ejection fraction, where inflammation significantly contributes to pathophysiology, and in post-operative cardiovascular patients, where inflammation drives complications.

### Direct anti-hypertrophic and anti-fibrotic actions

At the molecular level, vitamin D directly targets key drivers of pathological cardiac remodeling. It suppresses cardiac fibroblast proliferation by inhibiting the TGF-β1/Smad3 axis while concurrently activating SIRT1 signaling pathways [23]. These concerted actions attenuate the dual processes of cardiomyocyte hypertrophy and interstitial fibrosis, thereby preserving myocardial compliance. By opposing these maladaptive structural changes, which are central to the progression of heart failure, particularly heart failure with preserved ejection fraction (HFpEF), vitamin D signaling helps maintain standard cardiac architecture and function [24]. This mechanistic role is supported by clinical observations showing that vitamin D deficiency exacerbates post-infarction remodeling, whereas supplementation attenuates these deleterious changes [25].

### Epidemiological and preclinical evidence: building the case

Population-based studies and animal research provide robust experimental support for the molecular evidence, forming the basis for subsequent clinical trials and informing early therapeutic expectations.

Epidemiological investigations across diverse global populations consistently demonstrate a graded inverse association between serum 25(OH)D concentrations and the incidence of significant CVD events [25]. Observational evidence consistently links higher vitamin D status with improved cardiovascular outcomes. Meta-analyses demonstrate that individuals with 25(OH)D concentrations in the highest quartile exhibit significantly reduced risks of coronary heart disease and hypertension relative to those in the lowest quartile [26]. In a large collaborative analysis by the Emerging Risk Factors Collaboration, EPIC-CVD, and the Vitamin D Studies Collaboration, non-linear Mendelian randomisation analyses in over 386,000 participants revealed that genetically predicted 25(OH)D concentrations showed an inverse association with mortality outcomes at very low levels but, across the full range of vitamin D status, no significant causal effect on coronary heart disease, stroke, or all-cause mortality was observed [27]. These findings temper earlier causal claims and underscore the need for threshold-based approaches.These associations generally persist after adjustment for conventional risk factors, though the potential for residual confounding remains. Preclinical studies substantiate the biological plausibility of these associations: genetically modified VDR-knockout models consistently develop hypertension and cardiac hypertrophy, accompanied by dysregulated activation of the renin-angiotensin-aldosterone system [28]. At the same time, diet-induced deficiency recapitulates key aspects of human vascular pathology, including endothelial dysfunction and accelerated atherosclerosis [29].

The reversibility of these phenotypes with vitamin D repletion strengthens the case for a causal role, while simultaneously highlighting that the timing of intervention may be critical, potentially favoring prevention over reversal of established disease.

### The clinical trial landscape: emergence of a paradox

The translation of compelling preclinical and epidemiological signals into definitive clinical testing has yielded a persistent and intellectually challenging paradox. As detailed below, large-scale RCTs have uniformly failed to replicate the anticipated cardiovascular benefits, necessitating a critical reappraisal of both experimental models and trial paradigms. Despite ro(bust preclinical evidence supporting a role for vitamin D in cardiovascular regulation, large-scale RCTs have consistently failed to demonstrate significant reductions in hard cardiovascular endpoints following supplementation in unselected populations.The VITAL trial reported no statistically significant effect of vitamin D supplementation on the primary composite outcome of major adverse cardiovascular events—defined as myocardial infarction, stroke, or cardiovascular death—among generally healthy adults [9, 30]. Similarly, the ViDA study in New Zealand, which evaluated monthly high-dose vitamin D (100,000 IU) in older individuals, observed no meaningful reduction in all-cause mortality or hospitalization rates, although secondary analyses suggested potential benefits for musculoskeletal outcomes such as falls and fractures [31]. Parallel findings emerged from the Australian D-Health trial, which employed an identical dosing regimen and likewise reported neutral effects on most cardiovascular endpoints; a modest signal for reduced heart failure incidence was noted but requires validation in hypothesis-driven studies powered for this specific outcome [32].

These findings are reinforced by a definitive individual participant data meta-analysis of 21 RCTs involving over 83,000 participants, which concluded that vitamin D supplementation does not lower the risk of major adverse cardiovascular events in the general population [33]. This collective body of evidence suggests that the null findings represent a genuine lack of effect rather than a type II statistical error (Table 1). Nevertheless, aggregate null results may obscure meaningful heterogeneity in treatment response across biologically distinct subgroups. Post hoc analyses from several major trials provide quantitative support for subgroup-specific benefits. In the ViDA study, individuals with

**Table 1:** Vitamin D Supplementation and Cardiovascular Outcomes: Summary of RCTs

Trial / Study	VITAL Manson et al., [30]	ViDA Scragg et al., [31]	D-Health Thompson et al., [32]	Barbarawi et al. Meta-Analysis [33]
Population & Key Characteristics	*N* = 25,871; men ≥ 50, women ≥ 55; generally healthy U.S. adults without prior CVD or cancer	*N* = 5,110; community-dwelling adults aged 50–84 years, New Zealand	*N* = 21,315; adults aged 60–84 years, Australia	*N* = 83,291 (pooled from 21 RCTs); diverse populations including general community and selected risk groups
Inclusion/Exclusion Summary	Excluded: prior CVD, cancer, renal failure, hypercalcemia, other major illnesses	Excluded: current treatment for CVD, renal impairment (creatinine > 150 µmol/L), hypercalcemia, recent supplementation	Excluded: current high-dose vitamin D use (> 500 IU/day), hypercalcemia, renal failure, sarcoidosis	Inclusion: RCTs reporting CVD outcomes with vitamin D vs. placebo; no restriction by baseline deficiency status
Intervention (Dose & Regimen)	Cholecalciferol 2,000 IU/day (and/or omega-3 fatty acids); placebo-controlled	Cholecalciferol 100,000 IU/month (oral bolus); placebo-controlled	Cholecalciferol 60,000 IU/month (oral bolus); placebo-controlled	Various: daily (400–5000 IU), weekly, or monthly bolus regimens
Baseline 25(OH)D Status	Mean ~ 30 ng/mL (majority sufficient)	Mean ~ 27 ng/mL (27% <20 ng/mL)	Mean ~ 24 ng/mL (10% <20 ng/mL)	Variable across trials; not selected for deficiency in most
Primary Cardiovascular Endpoint	MACE (composite of MI, stroke, CV death)	CVD events (MI, stroke, CV death, heart failure, revascularization)	MACE (composite of MI, stroke, CV death)	MACE (composite, as defined by each trial); also MI, stroke, CV mortality, all-cause mortality
Key Cardiovascular Findings	HR 0.97 (95% CI, 0.85–1.12); *p =* 0.66	HR 1.02 (95% CI, 0.87–1.20); *p =* 0.81	HR 0.91 (95% CI, 0.81–1.01); *p =* 0.08	RR for MACE: 1.00 (95% CI, 0.95–1.06); *p =* 0.85
Comments	No selection for deficiency; null primary outcome. Subgroup analysis suggested possible benefit in those with normal BMI	Monthly bolus regimen; null primary outcome. Secondary analysis showed improved pulse wave velocity in deficient participants (*p =* 0.03)	Signal for reduced MI in prespecified analysis (HR 0.81, 0.67–0.98). Monthly bolus design may limit generalizability to daily dosing	Largest meta-analysis to date. Consistent null findings across all prespecified subgroups (by dose, baseline 25(OH)D, intervention duration, and study quality)

**Abbreviations: **25(OH)D, 25-hydroxyvitamin D; BMI, body mass index; CI, confidence interval; CV, cardiovascular; CVD, cardiovascular disease; HR, hazard ratio; MACE, major adverse cardiovascular events; MI, myocardial infarction; RR, risk ratio

**Note: **All trials primarily enrolled participants from the general community without pre-selection for vitamin D deficiency. The consistent neutral findings across these large-scale studies do not support the routine use of vitamin D supplementation for cardiovascular protection in the general population

suboptimal baseline vitamin D status exhibited improved arterial hemodynamics, as evidenced by reduced pulse wave velocity, enhanced arterial compliance, and favorable modulation of vascular surrogate endpoints(HR:-5.7%; 95% CI:-10.8, -0.6, *p* = 0.03) [31]. Subgroup analyses stratified by glycemic status revealed that both vitamin D insufficiency and deficiency were linked to higher mortality and adverse cardiovascular outcomes in individuals with prediabetes as well as those with established type 2 diabetes. Dose-response modeling further demonstrated a nonlinear relationship between serum 25(OH)D concentration and risk, with the nadir for both all-cause and cardiovascular mortality occurring near 60 nmol/L (1 nmol/L = 0.4 ng/mL). Notably, concentrations exceeding this threshold showed a modest upward inflection in risk [34]. These signals, though derived from exploratory analyses, highlight the potential for targeted repletion in deficient or metabolically dysregulated subgroups.

Emerging epigenetic insights further suggest that molecular responsiveness to vitamin D may be mediated by epigenetic reprogramming. For instance, vitamin D supplementation in acute coronary syndrome patients (*n* = 125) induces activation of VDR and downregulates Mir361-5p, correlating with improved left ventricular ejection fraction at 6 months (*p* = 1.1 × 10⁻⁴). Compared with standard treatment plus vitamin D supplementation, standard treatment alone was associated with higher incidences of heart failure (HF) and MACE, as well as a significantly greater proportion of patients with recurrent MACE [35]. In recognition of this biological complexity, recent expert consensus statements advocate for a precision-based approach to repletion. The Italian National Institute for Cardiovascular Research, for example, proposes a risk-stratified algorithm that integrates baseline 25(OH)D status, body mass index, renal function, inflammatory burden, and genetic variants in vitamin D metabolism pathways to inform dosing intensity and monitoring frequency [36].

While conceptually compelling, the clinical implementation of such frameworks remains limited by the absence of standardized assays for bioavailable or free 25(OH)D, validated polygenic risk scores, and health economic evaluations demonstrating cost-effectiveness. This translational gap directly reflects the fundamental limitations in conventional trial design that obscure vitamin D’s cardiovascular effects—specifically, the inclusion of vitamin D–replete participants, fixed-dose regimens ignoring interindividual pharmacokinetic variability, lack of stratification by functional biomarkers, and insufficient follow-up to capture vascular remodeling processes. Future investigations must therefore move beyond “one-size-fits-all” supplementation paradigms toward adaptive, biomarker-guided, and genetically informed intervention strategies capable of identifying and targeting responsive endotypes.

## Deconstructing the discrepancy: interpreting neutral trial results

The gap between biological plausibility and clinical trial outcomes requires critical examination. Potential explanations range from methodological limitations to fundamental biological considerations that may account for this paradox.

### Methodological limitations of RCTs

A fundamental methodological constraint in interpreting the essentially null results of major vitamin D trials pertains to their participant selection and intervention design. Many RCTs enrolled populations with baseline 25(OH)D concentrations at or above sufficiency thresholds, creating a “floor effect” whereby supplementation offers minimal additional benefit [37]. This design inherently contrasts with observational data that identify elevated cardiovascular risk predominantly at the lower extreme of the 25(OH)D distribution. Consequently, investigators may not have powered these trials adequately to detect cardiovascular benefits specifically conferred by correcting severe vitamin D deficiency. Further complicating the evidence synthesis is significant heterogeneity across studies in dosing strategies—ranging from daily low-dose to intermittent high-dose bolus regimens—and total intervention duration, which may be insufficient to reverse decades-long vascular pathology [38]. Moreover, the dosages employed, while adequate for skeletal health, may be suboptimal for eliciting cardiovascular effects, particularly in populations with altered vitamin D metabolism, such as individuals with obesity [39].

The collective impact of these factors—coupled with varying endpoint definitions and the frequent underrepresentation of truly high-risk, deficient cohorts—substantially compromises the ability to detect a potential signal for cardiovascular risk reduction, should one exist [40].

### Biological complexity

The relationship between vitamin D status and cardiovascular health is intrinsically complex and non-monotonic, presenting a fundamental challenge for the design and interpretation of clinical trials. Evidence increasingly supports a U-shaped or threshold-dependent association, wherein both severe deficiency and sustained very high concentrations of 25(OH)D may elevate cardiovascular risk, deviating from an optimal intermediate range [41]. This complexity is compounded by significant modulation from host factors. Genetic variation plays a key role, as common polymorphisms in genes governing vitamin D metabolism (e.g., GC, CYP2R1) and signaling (e.g., VDR) contribute to substantial inter-individual differences in systemic 25(OH)D levels and tissue responsiveness [42].

Furthermore, the biological activity of vitamin D is modulated by a critical interplay with nutritional and metabolic status. Its efficacy is dependent on adequate magnesium, an essential cofactor for the enzymes involved in vitamin D metabolism; magnesium deficiency can therefore significantly blunt vitamin D signaling [43]. In cardiometabolic disorders, this nutrient interdependence is further complicated. Adipose tissue sequesters vitamin D, reducing its bioavailability in obesity, while states of insulin resistance, diabetes, and dyslipidemia create a backdrop of chronic inflammation and endothelial dysfunction that may override vitamin D’s beneficial pathways [44].

These multifaceted, context-dependent interactions suggest that the cardioprotective effects of supplementation might be too subtle to detect in conventional RCTs using rigid, single clinical endpoints. This underscores the importance of considering pharmacokinetic and pharmacodynamic (PK/PD) principles. The dosing regimen itself influences outcomes: comparative analyses indicate that daily or weekly administration achieves more stable 25(OH)D levels and greater parathyroid hormone suppression than monthly bolus dosing, which can produce transient supraphysiological peaks and potentially blunt endocrine effects [45].The need for tailored dosing is particularly evident in obesity, where vitamin D is sequestered in adipose tissue, altering its distribution and bioavailability. To achieve target serum concentrations in this population, doses 2–3 times higher than standard are often required. This is quantified by a pooled analysis of RCTs, which suggests that each 10 kg/m² increase in BMI may necessitate an additional 1,000 IU/day to attain comparable 25(OH)D levels [9]. Future trials should adopt adaptive dosing algorithms informed by baseline BMI and on-treatment 25(OH)D monitoring to ensure adequate bioavailable vitamin D across diverse metabolic phenotypes.

### Confounding and reverse causation

Multiple lifestyle and physiological factors—including nutritional intake, physical activity, and general health status—strongly influence circulating 25(OH)D concentrations [9]. Higher circulating 25(OH)D concentrations are frequently associated with lifestyle patterns characterized by increased sun exposure and nutrient-dense dietary intake—factors independently linked to reduced cardiovascular risk [46]. This association introduces considerable confounding in observational analyses, limiting causal inference. Furthermore, the relationship is likely bi-directional. The state of chronic illness can lead to reduced outdoor activity, diminished appetite, and altered metabolism, thereby lowering 25(OH)D levels—a phenomenon of reverse causation where disease suppresses vitamin D status rather than deficiency causing disease [47]. Varying lifestyles and differences in skin pigmentation across ethnic and geographic populations actively modulate the relationship between vitamin D status and cardiovascular disease risk, amplifying its contextual complexity [48]. Consequently, low vitamin D may often serve as a marker of systemic ill health, inflammation, and metabolic dysregulation, rather than a directly modifiable causal factor [49]. To disentangle this directionality, future research must prioritize longitudinal cohorts and Mendelian randomization studies [27].

### Integrative interpretation: reconciling mechanism with clinical trial evidence

The persistent divergence between preclinical efficacy and clinical neutrality underscores the fundamentally context-dependent nature of vitamin D biology. Its pleiotropic actions may confer cardiovascular protection only within a ‘permissive biological window’—defined by severe deficiency, heightened renin–angiotensin system activity, or pronounced systemic inflammation—conditions systematically underrepresented in general-population trials. This conceptual shift implies that future investigations must abandon universal supplementation paradigms in favor of strategies designed to identify and target biologically distinct ‘responsive endotypes’. Such an approach requires integration of genomic, metabolic, and inflammatory biomarkers a priori, rather than relying on post hoc exploratory analyses. A therapeutic window for efficacy may exist primarily in states of severe deficiency [50], heightened RAAS activation, or pronounced systemic inflammation [51], scenarios not adequately represented in the general populations enrolled in most major trials. To move beyond these neutral findings, a paradigm shift towards precision nutrition is imperative. This necessitates the application of omics-based stratification (e.g., genomics, transcriptomics) and the development of adaptive, phenotype-targeted dosing protocols to elucidate subgroup-specific effects. Furthermore, the emergence of novel bioinformatics frameworks now permits the dynamic modeling of the complex interplay between vitamin D status and cardiovascular pathophysiology, integrating multi-omics datasets to generate actionable, personalized insights [52].

### Future directions and clinical implications

This evidence gap does not end scientific inquiry but requires refined research questions and methodologies that address the complexities of vitamin D biology and cardiovascular pathophysiology.

### Toward precision nutrition and genomic stratification

A decisive shift from the universal supplementation paradigm toward a precision medicine framework is imperative for future investigations into vitamin D and cardiovascular health. This approach must account for the well-documented heterogeneity in vitamin D metabolism, transport, and tissue-level response. Operationalizing this vision requires a multi-parametric framework that integrates baseline 25(OH)D, functional genetic variants (e.g., in GC, CYP2R1, VDR), metabolic and inflammatory biomarkers (e.g., PTH, hs-CRP, IL-6), and clinical factors (e.g., BMI, renal function). A candidate genetic panel for initial stratification could include rs2282679 (GC), rs10741657 (CYP2R1), and rs1544410 (VDR) [42].

For high-risk individuals—such as those with confirmed biochemical deficiency (25(OH)D < 20 ng/mL) and a high genetic risk profile—a precision dosing algorithm is proposed. This may involve a loading phase (e.g., 25,000–50,000 IU weekly for 4–6 weeks) followed by a maintenance regimen (800–4,000 IU/day), aiming for on-treatment 25(OH)D levels of 30–50 ng/mL. Pharmacokinetic principles support daily or weekly dosing over monthly bolus regimens to minimize serum fluctuations and optimize tissue delivery. Building upon the established need for higher doses in obesity, an adiposity-adjusted dosing algorithm should be implemented (e.g., initial calculation based on BMI strata). Monitoring at 3–4 month intervals during titration is advised, with a safety threshold of 25(OH)D to avoid hypercalcemia [53, 54].

The identification of responsive subgroups will be further refined by integrating genetic data with clinical variables using machine-learning algorithms, which show increasing promise for predicting individual response profiles [55]. Concurrently, Mendelian randomization studies remain invaluable for refining causal inference and identifying potential effect thresholds [56]. Beyond monotherapy, emerging evidence supports the investigation of nutrient co-supplementation as a mechanistically grounded strategy; for instance, combining vitamin D and omega-3 fatty acids yields greater improvements in endothelial function and arterial stiffness than vitamin D alone [57].

To validate these precision approaches, future interventional trials must prioritize enrolling participants with confirmed deficiency to ensure a sufficient biological gradient [58]. Their endpoints should evolve beyond major adverse cardiovascular events to include more sensitive, pathophysiology-focused measures, such as advanced cardiovascular imaging and dynamic functional biomarkers, capable of capturing subtler yet clinically relevant effects [59]. Ultimately, the integration of genomic, transcriptomic, and epigenomic data holds the potential to advance the field from broad stratification to truly personalized vitamin D dosing strategies [60].

### Current clinical practice recommendations

Based on the consistent null findings from major randomized trials, current clinical guidelines do not endorse the routine measurement of serum 25(OH)D or vitamin D supplementation for the primary or secondary prevention of cardiovascular disease in the general population [7, 61]. The established indications for vitamin D assessment and repletion remain firmly rooted in skeletal health, as defined by evidence-based guidelines which recommend maintaining a serum 25(OH)D level of at least 20 ng/mL to support bone metabolism and prevent disorders such as osteoporosis [62].

To optimize cardiovascular outcomes, clinicians should prioritize guideline-directed, evidence-based interventions—including blood pressure and lipid management—over vitamin D supplementation, which remains indicated primarily for skeletal health: specifically, to preserve bone integrity and reduce fall risk in older adults and individuals with osteoporosis [63]. For patients in these categories, any potential cardiovascular effects arising from correcting the deficiency should be considered a secondary benefit rather than the primary therapeutic goal [9].

That said, clinicians should target vitamin D repletion in high-risk populations—particularly older adults and patients with chronic kidney disease, diabetes, or inflammatory disorders—in whom deficiency is prevalent and consistently associated with adverse health outcomes [36, 64]. Some meta-analyses suggest that optimizing vitamin D status in these specific cohorts may be associated with modest reductions in all-cause and cardiovascular mortality [28, 65]. However, this association does not imply a direct cardioprotective effect. Patient education materials and clinical guidelines should therefore emphasize that while maintaining vitamin D sufficiency supports general health, it is not an evidence-based approach for reducing cardiovascular risk. These insights collectively advocate for a shift from population-wide supplementation to a risk-adapted strategy, aligning with the broader paradigm of precision medicine in cardiometabolic care.

### Advancing causal inference: from target trial emulation to heterogeneous treatment effects

To bridge the gap between RCTs limitations and real-world complexity, rigorous observational studies have been increasingly adopted in nutritional epidemiology, utilizing advanced analytical approaches to minimize confounding. To address inherent limitations of conventional observational studies, target trial emulation (TTE) has emerged as a rigorous methodological framework for strengthening causal inference in nutritional epidemiology. As articulated by Fu (2023) in a Journal of the American Society of Nephrology methodology primer, TTE prospectively applies the defining components of a randomized controlled trial—eligibility criteria, treatment strategies, and outcome measures—to the analysis of observational data. This methodological approach is designed to mitigate immortal time bias and confounding by indication [66].

While current trial data do not yet support routine use of machine learning for individual-level prediction, emerging methods—including g-computation and meta-learners (e.g., X-learner)—offer a framework for transporting conditional average treatment effects (CATE) from trials like VITAL and D-Health to heterogeneous clinical populations [67]. Future integration of genomic data (e.g., GC, CYP2R1), dynamic biomarkers, and EHR-derived phenotypes may enable personalized estimation of benefit—for instance, identifying subgroups with severe deficiency and high genetic predisposition who stand to gain the most. Embedding such models into decision-support systems represents a scalable path toward evidence-based precision nutrition, though prospective validation remains essential.

## Conclusions

The evolution of vitamin D from a promising cardioprotective candidate to a focus of neutral clinical trial results provides an instructive paradigm for translational research. This trajectory not only illustrates the critical distinction between epidemiological associations and causative relationships but also reaffirms the necessity of rigorous randomized controlled trials for validating nutritional interventions. While the initial proposition of a simple vitamin-based solution for cardiovascular disease prevention remains unsubstantiated, this extensive research endeavor has significantly advanced methodological standards in nutritional epidemiology and trial design.

Nevertheless, the substantial body of molecular evidence remains compelling and warrants consideration of vitamin D’s potential role in cardiovascular homeostasis. The principal challenge now resides in identifying specific contextual determinants—whether defined by genetic predisposition, particular disease states, or distinct metabolic environments—wherein vitamin D optimization yields measurable cardiovascular benefits. Emerging data indicate that vitamin D exerts its most discernible protective effects in biologically defined high-risk subgroups—particularly individuals with severe deficiency, elevated systemic inflammation, or pronounced metabolic dysregulation [68].

Future investigations should move beyond conventional uniform supplementation paradigms and adopt adaptive, multi-omics–informed trial designs that can resolve biologically meaningful heterogeneity in cardiovascular responses to vitamin D, including the RAAS, oxidative stress and inflammatory pathways, and endothelial function networks.Until evidence from rigorously designed precision-oriented trials, vitamin D supplementation should remain restricted to its well-validated skeletal indications; any observed cardiovascular effects may be interpreted as context-dependent secondary outcomes rather than primary therapeutic objectives.

Collectively, the evolving body of evidence underscores a fundamental principle of translational research: biologically plausible mechanisms must be tested through carefully designed clinical studies before being translated into therapeutic recommendations.

## Data Availability

No datasets were generated or analysed during the current study.

## Abbreviations

1,25(OH)D: 1,25-dihydroxyvitamin D
25(OH)D: 25-hydroxyvitamin D
BMI: body mass index
CVD: Cardiovascular disease
CYP27B1: 1-α-hydroxylase
VDRs: vitamin D receptors
RAAS: Renin-angiotensin-aldosterone system
eNOS: endothelial nitric oxide synthase
NO: nitric oxide
ROS: reactive oxygen species
HFpEF: heart failure with preserved ejection fraction
RCTs: randomized controlled trials
MACE: major adverse cardiovascular events
HF: heart failure
TTE: target trial emulation
CATE: conditional average treatment effects
